# Diet quality and adherence to nutritional recommendations: a multicenter study based on caregiver-child dyads

**DOI:** 10.1590/0102-311XEN167925

**Published:** 2026-07-06

**Authors:** Nelson Hun, Carolina Saravia, Jenny Arteaga, Felipe González-Fernández, Caroline Yans, Víctor Sepúlveda, Claudia Villablanca, Nicole Lasserre-Laso, Daniela Robles-Tapia

**Affiliations:** 1 Facultad de Salud, Universidad Santo Tomás, Antofagasta, Chile.; 2 Facultad de Salud, Universidad Santo Tomás, Talca, Chile.; 3 Facultad de Salud, Universidad Santo Tomás, Santiago, Chile.; 4 Facultad de Salud, Universidad Santo Tomás, Puerto Montt, Chile.; 5 Facultad de Salud, Universidad Santo Tomás, Los Ángeles, Chile.

**Keywords:** Food Guide, Students, Schools, Guías Alimentarias, Estudiantes, Escuelas, Guias Alimentares, Estudantes, Escolas

## Abstract

In recent decades, overweight and obesity among school-aged children have steadily increased, accompanied by a marked rise in the consumption of processed and ultra-processed foods, highlighting the need to examine school dietary patterns. This study aimed to assess diet quality and adherence to nutritional recommendations among schoolchildren, based on caregiver-reported information, within caregiver-child dyads across seven municipalities in Chile. A cross-sectional, descriptive, and correlational study was conducted with a sample of 2,115 dyads. Associations between sociodemographic variables and categories of the *Global Diet Quality Index* were analyzed using chi-squared tests and multinomial logistic regressions, and relative risks were calculated to estimate the magnitude of these associations. Statistical significance was set at p < 0.05. Children attending subsidized private schools showed higher overall diet quality (p ≤ 0.001), while those enrolled in municipal schools were more likely to meet recommendations for fruits (p = 0.001), vegetables (p = 0.009), and legumes (p = 0.000). Preschool children demonstrated greater adherence to nutritional recommendations compared with those in primary and secondary education. In conclusion, diet quality and adherence to nutritional recommendations vary significantly according to school type and educational level, reinforcing the need for tailored strategies to promote healthy eating across educational stages and school contexts.

## Resumen

En las últimas décadas, se ha producido un aumento continuo del sobrepeso y de la obesidad entre los niños en edad escolar, junto con un aumento significativo del consumo de alimentos procesados y ultraprocesados, lo que pone de relieve la necesidad de examinar los patrones alimentarios en las escuelas. El objetivo de este estudio fue evaluar la calidad de la dieta y el cumplimiento de las recomendaciones nutricionales entre los escolares, basándose en la información proporcionada por sus cuidadores, en díadas cuidador-niño de siete ciudades de Chile. Se realizó un estudio transversal, descriptivo y correlacional con una muestra de 2.115 díadas. Se analizaron las asociaciones entre las variables sociodemográficas y las categorías del *Índice Global de Calidad Dietética* mediante pruebas de chi-cuadrado y regresiones logísticas multinomiales, y se calculó el riesgo relativo para estimar la magnitud de las asociaciones. Se adoptó un nivel de significación estadística de p < 0,05. Los niños que asistían a escuelas privadas subvencionadas mostraron una mayor calidad alimentaria general (p ≤ 0,001), mientras que los que asistían a escuelas municipales eran más propensos a cumplir las recomendaciones de consumo de frutas (p = 0,001), verduras (p = 0,009) y legumbres (p = 0,000). Los niños en edad preescolar mostraron un mayor cumplimiento de las recomendaciones nutricionales en comparación con los de educación primaria y secundaria. En conclusión, la calidad de la dieta y el cumplimiento de las recomendaciones nutricionales varían significativamente según el tipo de escuela y el nivel educativo, lo que refuerza la necesidad de estrategias personalizadas para promover una alimentación saludable según la etapa educativa y el contexto escolar.

## Resumo

As últimas décadas testemunharam o aumento sustentado no excesso de peso e obesidade entre crianças em idade escolar e um crescimento significativo no consumo de alimentos processados e ultraprocessados, destacando a necessidade de examinar os padrões alimentares escolares. Este estudo buscou avaliar a qualidade alimentar e a conformidade com as recomendações nutricionais em crianças em idade escolar com base em informações relatadas por seus cuidadores dentro das díades cuidador-criança em sete cidades do Chile. Foi realizado um estudo transversal, descritivo e correlacional com uma amostra de 2.115 díades. Associações entre variáveis sociodemográficas e categorias do *Índice Global de Qualidade da Dieta* foram analisadas usando testes qui-quadrado e regressões logísticas multinomiais. O risco relativo foi calculado para estimar a magnitude das associações. Um nível de significância estatística de p < 0,05 foi adotado. Crianças que frequentavam escolas particulares subsidiadas apresentaram maior qualidade alimentar geral (p ≤ 0,001), enquanto aquelas em escolas municipais tinham maior probabilidade de atender às recomendações de frutas (p = 0,001), vegetais (p = 0,009) e leguminosas (p = 0,000). Crianças em idade pré-escolar demonstraram maior adesão às recomendações nutricionais do que aquelas do ensino fundamental e médio. A qualidade da dieta e a adesão às recomendações nutricionais variam significativamente de acordo com o tipo de escola e o nível educacional, reforçando a necessidade de estratégias personalizadas para promover uma alimentação saudável de acordo com o estágio educacional e o contexto escolar.

## Introduction

Dietary quality is defined as the overall nutritional profile of foods habitually consumed and may positively or negatively influence health outcomes ^1^. During childhood and adolescence, it is a critical determinant of physical growth, cognitive development, and the establishment of habits that persist into adulthood ^2^. These formative years are particularly important, as dietary behaviors adopted during this stage can shape either protective or adverse health trajectories across the lifespan ^3^. Adequate childhood nutrition has been associated with improved academic performance ^4^, reduced risk of noncommunicable diseases such as obesity, type 2 diabetes, and hypertension ^5^ ^,^ ^6^, and enhanced cognitive and memory function ^7^.

In recent decades, dietary patterns have undergone a marked shift, characterized by declining consumption of fresh or minimally processed foods and a steady increase in the intake of ultra-processed products high in sugars, saturated fats, and sodium ^8^ ^,^ ^9^. This trend poses an urgent public health challenge, particularly in middle-income countries such as Chile, in which socioeconomic factors strongly influence food access and availability ^10^ ^,^ ^11^.

According to the *2024 Nutritional Map* published by the Chilean National Board of School Assistance and Scholarships (JUNAEB, acronym in Spanish) ^12^, 27% of Chilean schoolchildren are classified as overweight and 17.9% as having obesity, representing increases of 0.4 and 0.5 percentage points, respectively, compared with 2023. The prevalence of severe obesity remained stable at 6%. These findings highlight a concerning trend in the nutritional status of school-aged children and underscore the need to evaluate dietary quality in this population.

School-based evidence further emphasizes the influence of sociodemographic characteristics. Differences in adherence to healthy eating habits, particularly dairy and fruit consumption, have been documented according to school type, with higher adherence observed in private schools compared with municipal and subsidized institutions ^13^. Similarly, higher dietary quality has been reported among children living in urban areas than among those in rural settings ^10^ ^,^ ^14^. Additional evidence indicates that adherence to nutritional recommendations is greater among girls and among children who share meals with their families ^10^.

From a relational perspective, children’s diet quality depends not only on individual choices but also on structural and contextual factors. In this regard, caregiver-child dyads represent key units of analysis, in which caregivers’ perceptions, knowledge, and practices directly influence children’s food-related decisions. These interactions are shaped by socioeconomic determinants such as educational level, household income, and working conditions, all of which affect food availability and choices. These elements highlight the importance of analyzing eating practices not only at the individual level but also within the relational context of caregiver-schoolchild dyads, conceptualized as interactive units in which caregivers’ decisions, perceptions, and dietary behaviors directly shape those of the child ^15^.

In this study, dietary quality was assessed using the *Global Dietary Quality Index* (GDQI) ^1^, a validated instrument that captures the frequency of consumption of healthy and unhealthy foods, as well as meal patterns. Adherence to dietary recommendations was determined based on the *Chilean Dietary Guidelines*, enabling an integrated evaluation of both quality and adequacy of children’s diets as reported by their caregivers.

Based on this framework, this study aimed to evaluate dietary quality and adherence to nutritional recommendations among schoolchildren using caregiver-reported information within the framework of caregiver-schoolchild dyads across seven Chilean cities. Each dyad consisted of a primary caregiver and a schoolchild, enabling assessment of the child’s diet from the caregiver’s perspective. We hypothesized that schoolchildren attending municipal schools and those whose caregivers had lower educational attainment would demonstrate poorer overall dietary quality and lower adherence to dietary and nutritional recommendations compared with those attending subsidized private schools, regardless of sex and grade.

## Methods

### Participants

The study included 2,115 caregiver-child dyads, composed of mothers, fathers, and/or primary caregivers and their school-aged children residing in seven Chilean cities: Iquique, Antofagasta, Viña del Mar, Talca, Concepción, Los Ángeles, and Puerto Montt. Although adults completed the questionnaire, all data referred specifically to the children under their care. Consequently, the reported dietary patterns reflect the eating behaviors of school-aged children. The questionnaire included the GDQI ^1^ and collected sociodemographic information such as the child’s sex and age, the caregiver’s educational level, and household income.

The required sample size was estimated a priori using G*Power software, version 3.1.9.7 (http://www.gpower.hhu.de/), based on a chi-squared test of association between categorical variables. Parameters included a significance level of α = 0.05, statistical power of 1-β = 0.95, and an effect size of w = 0.10. This analysis indicated a minimum required sample of 1,977 dyads. The final sample (n = 2,115) exceeded this threshold, ensuring adequate statistical power for the planned analyses. Among caregivers responsible for children’s diets, 90.1% were women and 9.9% were men.

### Instruments

Dietary quality was assessed using the GDQI, an instrument developed in Chile and applied to school-aged populations ^1^ ^,^ ^10^. The tool comprises 12 items grouped into three dimensions: healthy foods, unhealthy foods, and meal timing. The healthy foods dimension assesses consumption of fruits, vegetables, legumes, dairy products, and fish; the unhealthy foods dimension captures intake of cookies, sweets and pastries, sugar-sweetened beverages, added sugar, and fried foods; and the meal timing dimension evaluates whether participants regularly consume breakfast, lunch, and dinner. Scores range from 0 to 120 points, with higher scores indicating better dietary quality. Results are categorized as healthy diet (90-120 points), diet requiring improvement (60-89 points), or unhealthy diet (< 60 points). Adherence to food group recommendations was determined according to the *Chilean Dietary Guidelines*, based on the minimum recommended intake frequency.

### Procedure

The study was introduced to participating schools nationwide, and digital questionnaires were subsequently distributed to caregivers via each institution’s official communication channels. Upon accessing the survey link, participants reviewed the study information and provided informed consent before completing the questionnaire. Data collection took place from May to October 2024.

### Statistical analysis

This study represents the first phase of data collection from a broader longitudinal project. Accordingly, the analyses presented are exploratory and based on a cross-sectional approach. The term “relative risk” is used in a comparative, non-causal sense to estimate the magnitude of the associations observed at this stage. Future publications will include longitudinal and multivariate analyses to assess changes over time and adjust for potential confounding variables.

Data were analyzed using IBM SPSS Statistics, version 25 (https://www.ibm.com/). Associations between sociodemographic variables and dietary quality categories were examined using chi-square tests. Relative risks (RR) and 95% confidence intervals (95%CI) were calculated to estimate the strength of these associations. Additionally, multinomial logistic regression models were performed controlling for caregiver educational level and household income. Statistical significance was set at p < 0.05. Spearman’s correlation was used to examine associations between dietary quality scores and continuous or ordinal sociodemographic variables, including caregiver age, educational level, and household income.

### Declaration of AI use

Artificial intelligence assisted exclusively with manuscript translation. The authors conducted the final review and assume full responsibility for the content.

### Ethical approval

The study was reviewed and approved by the Scientific Ethics Committee of Santo Tomás University (approval code 49-24). Written informed consent was obtained from all participants prior to enrollment.

## Results

### Sociodemographic characteristics

The study sample was drawn from seven Chilean municipalities: Iquique (9.1%), Antofagasta (39.5%), Viña del Mar (4.1%), Talca (11.4%), Concepción (2.4%), Los Ángeles (13.1%), and Puerto Montt (20.4%). Nearly half of the children (48.2%) attended municipal schools, while 51.8% were enrolled in subsidized private institutions. Boys represented 51.1% of the sample and girls 48.7%. By educational level, 17% were in preschool, 72.8% in primary school, and 10.3% in secondary school. Most caregivers were women (90.1%), with men accounting for 9.9%. Most households (78.7%) reported a monthly income of USD 1,085 or less. Regarding caregiver educational attainment, 45.6% had completed secondary education, 22.9% held a university degree, 19.8% had completed technical training, 7.3% had completed only primary education, and 4.3% held postgraduate qualifications.

### Dietary quality

Table 1 presents dietary quality, categorized according to the GDQI and stratified by sociodemographic characteristics. Significant differences were observed by city of residence: Puerto Montt exhibited the highest proportion of children classified in the healthy diet category, whereas Antofagasta had the lowest, with a gap exceeding 17 percentage points. No significant differences were detected by child sex or by caregiver employment status. However, a clear trend was observed by household income, with higher income associated with a greater proportion of healthy diets and a lower prevalence of unhealthy diets.

**Table 1:** *Global Diet Quality Index* (GDQI) among students according to caregiver-reported sociodemographic characteristics.

Characteristics	GDQI score (mean)	Unhealthy		Needs improvement		Healthy		p-value
		n	%	n	%	n	%	
Total	79.2	236	11.2	1,314	62.1	565	26.7	
Municipality								< 0.001
Iquique	76.2	26	13.5	125	64.8	42	21.8	
Antofagasta	76.2	124	14.9	542	64.9	169	20.2	
Viña del Mar	82.2	9	10.5	48	55.8	29	33.7	
Talca	79.0	20	8.3	167	69.0	55	22.7	
Concepción	84.1	3	6.0	30	60.0	17	34.0	
Los Ángeles	82.7	19	6.9	167	60.3	91	32.9	
Puerto Montt	83.2	35	8.1	235	54.4	162	37.5	
Student’s sex								0.765
Male	79.0	125	11.6	674	62.4	281	26.0	
Female	79.5	110	10.7	638	61.9	282	27.4	
No response	80.5	1	20.0	2	40.0	2	40.0	
Student’s grade level								< 0.001
Pre-kindergarten	84.5	11	7.9	73	52.5	55	39.6	
Kindergarten	83.2	12	5.4	138	62.4	71	32.1	
First grade	82.3	20	10.8	104	56.2	61	33.0	
Second grade	80.6	15	8.8	103	60.6	52	30.6	
Third grade	79.2	22	10.3	134	62.9	57	26.8	
Fourth grade	79.9	21	8.4	167	66.8	62	24.8	
Fifth grade	77.1	26	13.6	129	67.5	36	18.8	
Sixth grade	77.7	23	11.6	127	64.1	48	24.2	
Seventh grade	73.0	35	19.1	116	63.4	32	17.5	
Eighth grade	76.3	20	13.4	101	67.8	28	18.8	
1st year of high school	78.8	6	10.7	35	62.5	15	26.8	
2nd year of high school	77.8	8	14.0	30	52.6	19	33.3	
3rd year of high school	77.1	15	21.1	36	50.7	20	28.2	
4th year of high school	79.1	2	6.3	21	65.6	9	28.1	
Caregiver’s sex								0.159
Male	77.4	30	14.4	133	63.6	46	22.0	
Female	79.4	206	10.8	1,180	62.0	517	27.2	
No response	87.8	-	-	1	33.3	2	66.7	
Employment status								0.611
Self-employed	80.4	36	10.3	212	60.6	102	29.1	
Employed	79.0	120	11.4	651	61.8	282	26.8	
Retired	82.9	-	-	7	70.0	3	30.0	
Unemployed	78.2	17	12.6	92	68.1	26	19.3	
Homemaker	79.2	63	11.1	352	62.1	152	26.8	
Income level (USD)								< 0.001
< 545	76.9	120	13.7	585	66.6	174	19.8	
545-1,085	79.9	79	10.2	478	61.9	215	27.8	
1,085-1,630	82.0	16	7.4	125	57.6	76	35.0	
1,630-2,175	83.3	10	7.7	67	51.5	53	40.8	
2,175-2,720	81.1	4	9.1	25	56.8	15	34.1	
2,720-3,260	81.0	5	13.5	16	43.2	16	43.2	
3,260-3,800	78.8	1	9.1	7	63.6	3	27.3	
3,800-4,350	93.4	-	-	-	-	-	-	
> 4,350		-	-	3	30.0	7	70.0	

Table 2 shows the distribution of GDQI categories by type of educational institution and stage of education. Significant differences were observed for both variables. Children attending subsidized private schools were more likely to be classified in higher GDQI categories compared with their peers in municipal schools. Similarly, preschoolers demonstrated a greater concentration in higher GDQI categories relative to children in primary and secondary education.

**Table 2:** *Global Diet Quality Index* (GDQI) according to type of educational institution and stage of education.

Variables	Unhealthy		Needs improvement		Healthy		p-value
	n	%	n	%	n	%	
Type of institution							< 0.001
Municipal school	133	13.0	677	66.3	211	20.7	
Subsidized private school	103	9.4	637	58.2	354	32.4	
Stage of education							< 0.001
Preschool	23	6.4	211	58.6	126	35.0	
Primary education	182	11.8	981	63.7	376	24.4	
Secondary education	31	14.4	122	56.5	63	29.2	
Total	236	11.2	1,314	62.1	565	26.7	

Regarding the multinomial logistic regression analysis examining the association between dietary quality and school type, adjusted for caregiver educational level and household income, the overall model was significant (χ^2^ = 72.70; p < 0.001) and explained between 1.9% and 4.1% of the variance in dietary quality (Nagelkerke). Caregiver educational level (odds ratio - OR = 0.77; 95%CI: 0.64-0.92; p = 0.004) and household income (OR = 0.85; 95%CI: 0.73-0.99; p = 0.043) were inversely associated with the likelihood of having a “poor” dietary quality, while school type was not statistically significant (OR = 1.37; 95%CI: 0.96-1.97; p = 0.084). Similarly, higher caregiver educational level (OR = 0.83; 95%CI: 0.74-0.93; p = 0.001) and household income (OR = 0.88; 95%CI: 0.80-0.96; p = 0.004) were associated with lower odds of being in the “needs improvement” category.

Likewise, a multinomial logistic regression analysis was performed to examine the association between dietary quality and stage of education, adjusted for caregiver educational level and household income. The overall model was significant (χ^2^ = 93.64; p < 0.001) and explained 5.2% of the variance in dietary quality (Nagelkerke). Compared with secondary school students, preschoolers were significantly less likely to present a “poor” (OR = 0.26; 95%CI: 0.14-0.50; p < 0.001) or “needs improvement” diet (OR = 0.67; 95%CI: 0.46-0.99; p = 0.047). Higher caregiver educational level and household income were inversely associated with poorer dietary quality (p < 0.001; data not shown).

### Adherence to dietary recommendations by food groups and meal times

Analysis of adherence to dietary recommendations by sex revealed significant differences only for fruit consumption (p = 0.007; RR = 1.309; 95%CI: 1.091-1.571), with girls demonstrating higher adherence (28.2%) than boys (23.1%). A similar pattern emerged when stratifying by caregiver sex. Statistically significant differences were again observed exclusively for fruit consumption (p = 0.016; RR = 1.555; 95%CI: 1.082-2.234), with children of female caregivers showing greater adherence (26.3%) than those of male caregivers (18.7%).

When adherence to dietary recommendations was analyzed by type of educational institution (Table 3) and stage of education (Table 4), statistically significant differences were observed across most food groups and meal-time indicators. Students from municipal schools showed greater adherence to recommendations for vegetables, fruits, and legumes, whereas those from subsidized private schools had higher adherence to milk consumption but also higher intake of sugar-sweetened beverages, added sugars, and fried foods. Regarding stage of education, preschoolers demonstrated the highest adherence to recommendations for fruits, milk, and legumes, while secondary school students showed the lowest adherence across most food groups. Significant differences were found for vegetables, fruits, milk, legumes, sugar-sweetened beverages, and added sugars, indicating a progressive decline in dietary quality with advancing stage of education. No significant differences were observed for fish or main meals.

**Table 3:** Adherence to dietary recommendations by food group and mealtime according to type of educational institution, based on caregiver-reported information.

Variables	Municipal schools				Subsidized private schools				χ^2^	RR	95%CI
	Meets		Does not meet		Meets		Does not meet				
	n	%	n	%	n	%	n	%			
Food group											
Vegetables	219	21.4	802	78.6	172	15.7	922	84.3	0.001 *	0.683	0.548-0.852
Fruits	287	28.1	734	71.9	253	23.1	841	76.9	0.009 *	0.769	0.633-0.939
Milk	324	31.7	697	68.3	418	38.2	676	61.8	0.002 *	1.330	1.112-1.592
Legumes	563	55.1	458	44.9	391	35.7	703	64.3	0.000 **	0.452	0.380-0.539
Fish	218	21.4	803	78.6	231	21.1	863	78.9	0.894	0.986	0.800-1.215
Cakes and pastries	160	15.7	861	84.3	133	12.2	961	87.8	0.019 *	0.745	0.581-0.954
Sugar-sweetened beverages	279	27.3	742	72.7	428	39.1	666	60.9	0.000 **	1.709	1.423-2.053
Added sugar	205	20.1	816	79.9	299	27.3	795	72.7	0.000 **	1.497	1.222-1.834
Fried foods	230	22.5	791	77.5	430	39.3	664	60.7	0.000 **	2.227	1.841-2.694
Mealtime											
Breakfast	782	76.6	239	23.4	858	78.4	236	21.60	0.312	1.111	0.906-1.363
Lunch	809	79.2	212	20.8	907	82.9	187	17.1	0.031 *	1.271	1.022-1.581
Dinner	731	71.6	290	28.4	783	71.6	311	28.4	0.990	0.999	0.827-1.207

95%CI: 95% confidence interval; RR: relative risk.

Note: statistical significance according to p-value: * p < 0.05; ** p < 0.001.

**Table 4:** Adherence to dietary recommendations by food group and mealtime according to stage of education, based on caregiver-reported information.

Variables	Preschool				Primary education				Secondary education				χ^2^
	Meets		Does not met		Meets		Does not meet		Meets		Does not meet		
	n	%	n	%	n	%	n	%	n	%	n	%	
Food group													
Vegetables	78	21.8	280	78.2	285	18.6	1,248	81.4	27	12.5	189	87.5	0.021 *
Fruits	124	34.6	234	65.4	382	24.9	1,151	75.1	32	14.8	184	85.2	0.000 **
Milk	177	49.4	181	50.6	506	33.0	1,027	67.0	57	26.4	159	73.6	0.000 **
Legumes	175	48.9	183	51.1	713	46.5	820	53.5	63	29.2	153	70.8	0.000 **
Fish	81	22.6	277	77.4	334	21.8	1,199	78.2	33	15.3	183	84.7	0.072
Cakes and pastries	43	12.0	315	88.0	207	13.5	1,326	86.5	40	18.5	176	81.5	0.077
Sugar-sweetened beverages	151	42.2	207	57.8	472	30.8	1,061	69.2	79	36.6	137	63.4	0.000 **
Added sugar	104	29.1	254	70.9	332	21.7	1,201	78.3	65	30.1	151	69.9	0.001 *
Fried foods	126	35.2	232	64.8	456	29.7	1,077	70.3	73	33.8	143	66.2	0.089
Mealtime													
Breakfast	289	80.7	69	19.3	1,186	77.4	347	22.6	159	73.6	57	26.4	0.133
Lunch	295	82.4	63	17.6	1,239	80.8	294	19.2	174	80.6	42	19.4	0.774
Dinner	267	74.6	91	25.4	1,097	71.6	436	28.4	145	67.1	71	32.9	0.158

Note: statistical significance according to p-value: * p < 0.05; ** p < 0.001.

Analyses examining associations between schoolchildren’s dietary quality and caregiver sociodemographic characteristics revealed no significant correlation between maternal age and dietary quality (Spearman’s rho = -0.009; p = 0.535). In contrast, dietary quality was positively and significantly associated with maternal educational attainment (Spearman’s rho = 0.154; p < 0.001). A similar positive and significant association was observed for household income (Spearman’s rho = 0.155; p < 0.001), indicating that higher socioeconomic resources were linked to better dietary quality among school-aged children (data not shown).

## Discussion

This study aimed to evaluate dietary quality and adherence to nutritional recommendations among schoolchildren using caregiver-reported information within caregiver-schoolchild dyads across seven Chilean cities. We hypothesized that children attending municipal schools and those whose caregivers had lower educational attainment would demonstrate poorer overall dietary quality and lower adherence to nutritional recommendations than their peers in subsidized private schools, independent of sex and grade level.

Our results support this hypothesis. Schoolchildren enrolled in subsidized private institutions were more likely to be classified in higher GDQI categories than those attending municipal schools. This finding is consistent with previous evidence showing lower overall dietary quality among students in municipal schools compared to those in subsidized and private institutions ^13^. A plausible explanation is that municipal schools predominantly serve children from socioeconomically disadvantaged backgrounds ^16^, in which limited household resources may restrict access to more nutritional foods ^17^. Consistent with this interpretation, higher household income has been repeatedly associated with improved access to and consumption of healthier foods ^18^ ^,^ ^19^ ^,^ ^20^.

The broader food environment also appears to contribute to these disparities. Research has documented an unequal distribution of food outlets, with municipal schools more frequently surrounded by vendors offering unhealthy products compared with subsidized and private institutions ^21^.

The finding that children attending municipal schools reported higher adherence to specific dietary recommendations - such as the consumption of fruits, vegetables, and legumes - despite exhibiting lower overall dietary quality scores suggests a complex nutritional dynamic. One possible explanation is the influence of the School Feeding Program (PAE, acronym in Spanish) ^22^, which is more consistently implemented in municipal schools and prioritizes foods aligned with national dietary guidelines, including unlabeled items and plant-based options. This may explain higher adherence to selected healthy foods. However, beyond school-provided meals, these children may be more exposed to unhealthy food environments characterized by limited access to nutritious options and a higher density of vendors offering ultra-processed products near public schools. Such contrasting environments - protective within the school setting but obesogenic in the surrounding context - may contribute to lower overall GDQI scores among municipal students, despite meeting certain specific dietary recommendations.

When examining GDQI scores by school level, preschoolers achieved higher scores than their peers in primary and secondary education, consistent with previous studies in Chilean school populations ^10^. This pattern may reflect closer dietary supervision by caregivers of preschool children, who have limited autonomy in food choices compared with older children ^23^. Caregiver food-related practices therefore emerge as an important influence at this stage ^24^.

Contrary to expectations, better dietary quality does not necessarily correspond to lower rates of overweight and obesity. National data indicate a sustained increase in these conditions across all school levels, particularly among preschool children ^12^. This discrepancy suggests the influence of additional determinants, including caregiver modeling behaviors, children’s exposure to food marketing, and the role of mass media ^25^.

Regional differences in dietary quality were also evident. Children in Puerto Montt had the highest proportion classified in the healthy diet category, whereas those in Antofagasta had the lowest, indicating a favorable trend in southern Chile. These results contrast with findings from the *Chilean National Food Consumption Survey* (ENCA, acronym in Spanish) ^19^, which reported higher prevalence of unhealthy diets in southern regions. However, ENCA data do not reflect more recent changes, such as the sustained increase in international migration in northern Chile, which may have influenced local dietary practices. Additionally, higher prevalence of food insecurity in northern regions, particularly in Antofagasta ^26^, together with geographic disparities in the availability and cost of fruits and vegetables - generally more affordable in central regions and less accessible in more remote areas - likely contributes to the observed variations ^27^ ^,^ ^28^.

Adherence to dietary recommendations also varied by school type. Children attending municipal schools reported greater adherence to recommendations for vegetables, fruits, and legumes, likely reflecting their access to PAE, which provides foods without warning labels, aligned with national dietary guidelines, and often includes an additional snack that increases exposure to healthier options ^22^. Conversely, adherence to milk consumption was higher among children in subsidized private schools (38.2%), possibly due to snacks brought from home. Evidence indicates that children from middle- and higher-income households are more likely to bring bread, fruit, and dairy products as snacks ^29^ ^,^ ^30^, consistent with the sociodemographic profile of students in these institutions.

Differences also emerged in adherence to recommendations regarding processed foods, including sugar-sweetened beverages, added sugars, and fried foods. Children attending subsidized private schools reported lower consumption of these items, consistent with studies showing a higher concentration of unhealthy food outlets surrounding municipal schools, particularly in low-income neighborhoods ^31^. While sugar-sweetened beverages are primarily consumed at home, the school environment may still shape children’s exposure to such products. Given that frequent consumption of ultra-processed foods has been associated with poorer academic performance ^7^ ^,^ ^31^, further research is needed to better understand the mechanisms underlying these disparities and to inform targeted interventions in the most vulnerable school contexts.

Consistent with previous research, preschool children demonstrated higher adherence to dietary recommendations than those in primary and secondary education ^23^ ^,^ ^24^. As children grow older, their autonomy over food choices and purchasing increases ^32^, parental oversight tends to decline, and the influence of peers and the broader school environment becomes more pronounced ^24^. Consequently, the widespread availability of unhealthy foods in school surroundings may hinder adherence to nutritional guidelines, particularly among older students ^31^.

The observed sex differences in dietary adherence - particularly the higher fruit consumption among girls - may reflect a combination of cultural, social, and familial factors. Although some studies have reported similar patterns, the evidence remain inconsistent, with other investigations showing conflicting results ^10^ ^,^ ^19^, suggesting that sex alone is unlikely to be a decisive determinant. One possible explanation is that girls are more frequently socialized into behaviors associated with self-care, self-regulation, and health consciousness, which may encourage healthier eating practices. In contrast, boys may be more strongly influenced by peer norms that associate certain foods - particularly ultra-processed items - with autonomy, strength, or culturally constructed notions of masculinity.

Within the family environment, female caregivers - who represented most of our sample - are often fundamental in food provision and the transmission of nutritional knowledge and culinary practices ^33^. This may translate into greater guidance or monitoring of children’s eating behaviors, especially among daughters. Evidence also indicates that when fathers participate in child feeding, they tend to align their practices with those of mothers ^34^, reinforcing established family patterns. These culturally and socially embedded dynamics may help explain sex-related differences observed in this study and highlight the importance of incorporating gender norms in future research.

Taken together, these findings underscore the central role of caregiver-schoolchild dyads, not only as a valuable approach for obtaining reliable dietary data but also as strategic targets for nutritional interventions aimed at promoting healthy eating among school-aged children ^24^ ^,^ ^34^.

This study has limitations. Its cross-sectional and correlational design precludes causal inference and limits the ability to assess changes over time. Additionally, the sample included only urban populations; therefore, the findings cannot be generalized to rural contexts. The use of non-probabilistic sampling further restricts the generalizability of the results to the broader Chilean school population. Moreover, since dietary data were reported by caregivers rather than directly observed or self-reported by children, reporting bias may have occurred, particularly for socially desirable behaviors such as healthy food consumption. Despite these limitations, the study included a large and geographically diverse sample, strengthening the analyses and enabling the identification of nationally relevant patterns. Overall, our results contribute valuable evidence to understanding dietary quality and adherence to nutritional recommendations in the school context and provide a foundation for designing interventions tailored to school type, educational level, and caregiver profile. Finally, these findings highlight the need to strengthen school-based nutrition interventions by aligning them more closely with existing public policies, such as Chile’s front-of-package warning label law and the PAE. Given the lower overall dietary quality observed among students in municipal schools, policymakers should consider reinforcing the nutritional scope and monitoring of PAE in vulnerable contexts, while expanding food education strategies that engage both caregivers and children. Additionally, educators should be supported with tools to integrate food literacy into the curriculum, helping students develop informed food choices beyond the school environment.

## Conclusion

Dietary quality and adherence to nutritional recommendations vary markedly by school type and educational level. Children attending subsidized private schools demonstrated higher overall dietary quality, whereas those in municipal schools were more likely to meet recommendations for specific healthy foods. Preschoolers exhibited greater adherence than their peers in primary and secondary education, underscoring the need for tailored strategies across educational stages and school contexts. These findings underscore the importance of considering the broader food environment and caregiver influences when designing school-based nutritional strategies. To enhance their effectiveness, such strategies should be aligned with existing public policies, including Chile’s front-of-package labeling law and the PAE. Strengthening these initiatives in socially vulnerable schools while equipping educators with tools to deliver food education may contribute to fostering healthier food practices among both children and caregivers.

## Data Availability

The research data are available upon request to the corresponding author.
